# The Potential of Fermentation-Based Processing on Protein Modification: A Review

**DOI:** 10.3390/foods14203461

**Published:** 2025-10-10

**Authors:** Negin Yousefi, Behdad Shokrollahi Yancheshmeh, Krist V. Gernaey

**Affiliations:** 1 Center for Process and Systems Engineering (PROSYS), Department of Chemical and Biochemical Engineering, Technical University of Denmark, 2800 Kongens Lyngby, Denmark; negyou@kt.dtu.dk (N.Y.); kvg@kt.dtu.dk (K.V.G.); 2 Research Group for Food Production Engineering, National Food Institute, Technical University of Denmark, 2800 Kongens Lyngby, Denmark

**Keywords:** fermentation, protein modification, techno-functional properties, digestibility, nutritional changes, sensorial properties

## Abstract

Proteins are fundamental to food systems due to their structural, nutritional, and functional roles. With increasing consumer awareness of health and sustainability, the demand for protein-rich foods from diverse and eco-friendly sources is rising. Fermentation has emerged as a transformative approach for enhancing the nutritional value, functionality, and sensory appeal of protein-based foods, while also contributing to environmental and economic sustainability. This review explores the multifaceted impact of fermentation on proteins, focusing on nutritional enhancement, functional improvements, technological adaptability, and sensory optimization. It highlights how microbial fermentation can modify protein structures, reduce allergenicity, improve digestibility, and generate bioactive compounds. The diversity of protein sources, microbial strains, and fermentation parameters offers a versatile platform for tailoring food products to meet evolving consumer expectations. By critically examining current research and industrial practices, this paper underscores the importance of selecting appropriate protein substrates and microbial hosts to maximize the benefits of fermentation. The insights provided aim to guide future innovations in developing sustainable, health-promoting, and consumer-acceptable fermented protein products.

## 1. Introduction

Fermentation is an ancient technique of food processing and preservation, involving the application of microorganisms (bacteria, molds, and yeasts) to obtain changes in the food. Since it is a natural method of food preservation, which raises the nutritional content and digestibility while lowering concentrations of antinutritional components, it offers a strong foundation to produce safe food with improved functionality and nutritional qualities [1,2]. The fermentation process starts with the inoculation of a substrate with the desired microorganism. The inoculated substrate is then held under environmental conditions suitable for its conversion into the desired fermented product. The crude product may be used directly, or it may be processed further to isolate specific molecular entities from it [3]. There is also a possibility where the fermented product is not consumed directly and stored for some reason. For example, *Lactobacillus plantarum*-fermented vegetables with pH control were microbiologically stable throughout 12 months of storage in hermetically sealed jars [4]. During fermentation, microorganisms can significantly affect protein structure through enzymatic activity. Proteases and peptidases produced by bacteria and fungi break down large protein molecules into smaller peptides and free amino acids, which can enhance digestibility and bioavailability. Microbial metabolism can also modify protein conformation, solubility, and functional properties, influencing texture, water-holding capacity, and emulsification in the final product. These structural changes not only improve nutritional quality but also contribute to the sensory and technological characteristics of fermented foods.

Fermentation involves the bioprocessing of food products using microorganisms and their enzymes to achieve desirable quality attributes. While it is often associated with anaerobic energy metabolism, not all fermentation processes are strictly anaerobic; some, such as certain lactic acid or acetic acid fermentations, can occur under microaerophilic or even aerobic conditions. Fermentable foodstuffs include those of both plant and animal origin, and fermenting microorganisms encompass a wide variety of yeasts, molds, and bacteria. Microbial metabolism and extracellular enzymes play a major role in modifying the composition, nutritional value, and functional properties of fermented foods [5].

The publication of numerous research papers about the effects of fermentation on the nutritional, sensorial, and physicochemical characteristics of different protein sources such as soybeans, lupins, chickpeas, peas, lentils, faba beans, and beans demonstrates the increasing interest in fermented protein-rich foods [6]. The present review article comprehensively compiles scientific knowledge on the influence of fermentation on various protein sources. In this paper, after briefly describing the properties of fermentation and proteins, we shed light on the effects of different fermentation methods on protein products and protein composition, in addition to the nutritional, physicochemical, functional, technological, antimicrobial, and sensorial properties.

## 2. Fermentation Processes and Products

Many key factors are required for fermentation, the most significant of which are the food ingredients, the moisture content, and the selected microorganisms. Furthermore, several process steps are usually required, including physical, thermal, and biological operations that are applied in a successive and strictly regulated way. Among the microorganisms, bacteria, yeasts, and molds are encountered as functional microorganisms in food fermentation. Among the bacteria, lactic acid bacteria are broadly used in domestic and industrial processing of fermented dairy, vegetable, meat, and cereal products. *Saccharomyces cerevisiae* (brewer’s or baker’s yeast) is the most important yeast for the production of alcoholic drinks and bread leavening. *Penicillium*, *Aspergillus*, and some of the *Mucorales* are broadly utilized to produce various products including wine, cheese, and soybean foods. Extracellular enzymes and microbial metabolism have profound effects on fermented food composition, as they decompose the food macromolecules (polysaccharides, lipids, and proteins) into smaller substances (dextrins, peptides, sugars, free fatty acids, and amino acids) and accumulate a range of metabolites in the process (acids, esters, alcohols, vitamins, aldehydes, and ketones) [7].

## 3. Types of Fermentation

Most commercial fermentations can be categorized into two groups: solid-state fermentation (SSF) and submerged fermentation (SmF). In SSF, microbes grow on a moist solid substrate with little or no free water, although capillary water may be present [8]. This method is traditionally used in the production of foods such as cheese, bread, and coffee. By contrast, SmF employs either a dissolved substrate (e.g., sugar solution) or a solid substrate suspended in sufficient water to form a slurry and is widely applied in the manufacture of products such as penicillin, beer, and recombinant insulin. While both techniques are valuable, they differ in advantages and limitations: SSF generally requires less water, energy, and processing equipment, and it often yields higher concentrations of certain secondary metabolites and enzymes; however, it is more difficult to scale up and control parameters such as temperature, aeration, and moisture content. SmF, on the other hand, provides better control of environmental conditions, reproducibility, and ease of scale-up in large bioreactors, making it the dominant method in industrial biotechnology, but it usually incurs higher operational costs and may result in more dilute product streams. These differences explain why SmF is preferred for large-scale industrial applications, while SSF remains advantageous in producing traditional fermented foods and specialized bioactive compounds [9].

SSF is suitable for microorganisms that tolerate lower moisture levels (typically 12–70%). Filamentous fungi such as *Aspergillus niger* and *Rhizopus oligosporus* grow well under these conditions, with optimal activity at 50–60% moisture and water activity (aw) of 0.93–0.96. By contrast, bacteria usually require high aw (>0.98) and are therefore better suited to SmF, which maintains near-saturated moisture conditions (aw ≈ 1.0). Yeasts are more adaptable and can grow in both systems, though they often achieve faster growth in SmF due to readily available dissolved nutrients. SmF also facilitates downstream purification of products [9]. Industrially, fungi dominate SSF applications such as oriental foods, composting, and ensilage, while bacteria and yeasts are widely used in SmF for large-scale food, beverage, and pharmaceutical fermentations. Overall, SSF offers higher product concentration with lower water and energy inputs but is harder to control at scale, whereas SmF allows better process monitoring and scalability but produces more dilute product streams [10].

Fermentation can be categorized into distinct stages based on the desired end product and the microorganisms involved. Initially, microbes acclimate to the substrate, releasing hydrolytic enzymes that begin breaking down complex macromolecules. During this adaptation phase, microbial proteases start converting proteins into peptides and free amino acids, thereby increasing nitrogen availability. In the primary fermentation stage, dominant metabolic pathways take over: in alcoholic fermentation, yeast enzymes break down carbohydrates into ethanol and carbon dioxide, while also releasing proteases that influence protein solubility and functionality. In lactic acid fermentation, lactic acid bacteria transform carbohydrates into lactic acid, lowering the pH, which promotes protein denaturation and improves digestibility. Heterolactic fermentation involves the breakdown of glucose into lactic acid, ethanol, and carbon dioxide, creating a mixed acid environment that further alters protein structures through acidification and enzymatic action. During the maturation phase, prolonged fermentation and microbial succession lead to the accumulation of bioactive peptides with antioxidant, antihypertensive, or antimicrobial properties, resulting from continued proteolysis. Each stage of fermentation not only modifies carbohydrates but also uniquely effects proteins through hydrolysis, denaturation, and the formation of functional peptides [11].

## 4. Fermentation Effects in the Food Industry

During fermentation, a variety of chemical/biochemical transformations occur, which are mainly dependent on the type of raw material and its quality, the applied fermentation stages, the microorganisms employed, and the type of final products. Numerous substances are produced from food compounds (proteins, lipids, and carbohydrates) during fermentation, which unequally contribute to the flavor of fermented foods.

## 5. Proteins

Proteins, the products of amino acid polymerization, are regarded as an essential component and structural element in foodstuffs. They exist as enzymes, hormones, and structural elements like cell walls and muscles. Moreover, they are a source of energy for plants and animals. They also play important roles in enhancing the textural, sensorial, functional, and nutritional properties of foods [12].

Proteins have numerous key functions in biological and food systems. They are tremendously complicated polymers, and their functional diversity is primarily owing to their conformation. The functional characteristics of proteins in foods pertain to their physicochemical and structural properties. If we are going to enhance the protein performance in food systems and elevate the applications of underutilized proteins like whey and plant proteins in both processed and traditional foods, it is necessary to deepen our understanding of the functional and physicochemical characteristics of proteins and the changes made to them during processing [13]. Although proteins play a number of non-nutritional roles in food products, the development and/or stabilization of food structures is their most common role. The general contribution of proteins/peptides to health and nutrition, in particular in terms of bioactivities, is so important because they have specific biological activities that can influence health positively. Due to their significant benefits, there is a trend in the food industry to develop products that are specifically designed to address health issues associated with proteins and peptides. Consequently, a great deal of attention should be paid to the retention of bioactive/nutritional attributes of proteins when they are utilized for the development of food structures. At the same time, it is vital to also address the disadvantages of the presence of proteins in food, for example, allergies, digestive issues, caloric density, nutrient imbalance, cost and environmental effects [14,15,16,17].

## 6. Effect of Fermentation on Protein Composition

Fermentation significantly improves protein quality by increasing crude and soluble protein levels, partly due to microbial protein synthesis and the selective consumption of carbohydrates and lipids, which raises the relative protein content [18,19]. Microbial proteases break down large, insoluble proteins into smaller peptides and amino acids, enhancing solubility, digestibility, and functional properties by exposing hydrophobic and charged groups. Fermentation also reduces crude fiber and anti-nutritional factors such as tannins, phytates, and trypsin inhibitors, improving mineral bioavailability and protein digestibility. Structural changes from acidification and enzymatic hydrolysis improve emulsifying and foaming properties. Additionally, proteolysis generates bioactive peptides with antioxidant activity, often linked to residues like histidine, cysteine, and aromatic side chains. Overall, fermentation reshapes protein composition, structure, and functionality through microbial metabolism and enzymatic action [19].

Fermentation can alter ash content, but the direction and extent of change depend on the substrate and fermentation conditions. For example, solid-state fermentation (SSF) of carob kibbles with *Pleurotus ostreatus* showed an initial decrease in ash due to microbial mineral uptake, followed by a modest increase by day 30, slightly exceeding the initial 3.5% *w*/*w* dry matter [20]. In maize flour co-fermented with *Lactiplantibacillus plantarum* and *S. cerevisiae*, ash content rose from 2.12% to 3.73% over 48 h. Conversely, cereal-based fermentations such as cocoyam flour and composite blends (e.g., sorghum–amaranth) often show a reduction in ash, likely due to mineral uptake or leaching [21]. These variations suggest that ash content changes during fermentation are context-dependent, influenced by microbial activity, substrate composition, and processing dynamics. Therefore, claims regarding ash modification should be supported by system-specific data [22].

Obadina et al. [23], investigated the impact of fermentation on the nutritional value and chemical composition of the fermented soymilk product *Nono*. They realized that the moisture, fat, and carbohydrate contents, respectively, lowered from 93.45 to 92.70, 2.18 to 0.87, and 1.52 to 0.60%, whereas the total solids, protein, and ash contents rose from 6.55 to 7.30, 2.62 to 5.09, and 0.23 to 0.74%, respectively. Their findings demonstrated that the calcium, magnesium, and iron contents in the fermented soymilk were elevated from 52.86 to 71.43, 7.66 to 8.87, and 28.00 to 40.00 mg/L, respectively, during the first 54 h of fermentation and subsequently diminished to 65.00, 7.83, and 28.00 mg/L, respectively, at the end of the process. On the other hand, the zinc content was raised from 4.42 to 6.75 mg/L in the entire process. It was found out that the protein, magnesium, calcium, iron, and zinc contents increased throughout the natural fermentation of soymilk. It was also revealed that the moisture content declined with the increase in the fermentation time, which can be attributed to the rise in the dry matter content due to the proliferation of the microbial cells. As the process progressed, the carbohydrate content of the fermented soymilk was reduced. This may be due to the action of the fermenting bacteria, which devoured the carbohydrates and converted them into the energy necessary for development and other cellular functions [23]. Omojasola [24], also reported a decline in moisture content in soymilk fermented for 30 h. As the fermentation progressed, moisture content diminished, whereas the total solid content increased during the process [24].

Fermentation almost restrained the activity of trypsin inhibitor in cowpeas. Therefore, fermentation was more efficient in eliminating the inhibitor than the other examined treatments (soaking, germination, and cooking) [25]. It has been claimed that SSF was able to raise the crude protein content and protein solubility in soy protein fermented with *B*. *licheniformis* YYC4, because of the production of microbial proteins and the consumption of the other nutrients in the substrate. *B. licheniformis* YYC4 is highly capable of producing protease which can decompose insoluble macromolecular proteins into soluble micromolecular proteins, peptides, and even free amino acids during fermentation [26].

Additional research has shown that after fermentation, the protein content increases. In research conducted on the *Lb. plantarum* fermentation of wheat flour enriched with pea protein, increments were discovered in the levels of protein, ash, and fat, probably owing to increased bacterial biomass and carbohydrate loss during the process [27]. Gantumur et al. [28], reported variations in the ash content of whey protein concentrate during fermentation with *S. cerevisiae* [28]. Arise et al. [29], mentioned that fermentation of Bambara nut or *Vigna subterranea* (L.) *Verdc*. protein isolates increased the protein yield and the ash content relative to the raw sample. In terms of the nutritional composition, the fermented sample had the highest ash and protein contents [29].

Kasprowicz-Potocka et al. [22], investigated the impact of a 24 h fermentation of lupin seeds using various yeast species on the chemical composition of the seeds. They stated that the mass fraction of proteins was increased following the fermentation, and their in vitro biological activity and digestibility were remarkably enhanced. Furthermore, the mass fraction of oligosaccharides and phytate decreased significantly, but not that of alkaloids. The pH value of the fermented samples declined due to the increase in the mass fractions of propionic and lactic acids. Fermentation using the Fermivin 7013 strain and *Saccharomyces cerevisiae* made the most desirable changes in the blue lupin seeds’ chemical composition [22].

The protein and moisture contents of maize protein flour rose through fermentation with *Saccharomyces cerevisiae* and *Lactobacillus plantarum*. The content and digestibility of the protein was most enhanced when *Lactobacillus plantarum* was used. The elevated protein content of the fermented maize flour can also be due to the reduction in the carbon levels of the total mass and the improvement of the cell biomass and the production of non-protein nitrogen containing substances such as amino acids, ammonia, amines, and peptides, as all of them are part of the crude protein content [30]. During fermentation, microbes consume carbohydrates as a source of energy and make carbon dioxide as a by-product, increasing the concentration of nitrogen in the fermented product; hence, the protein percentage in the remaining overall mass is increased [31].

Furthermore, fermentation significantly decreased the inhibitors of phytate, trypsin, and tannin. Similarly, the fiber and fat contents of maize flour diminished during fermentation. The reduction in the crude fiber content can be ascribed to the extracellular enzymes secreted by the microorganisms which partly hydrolyze and metabolize insoluble polysaccharides [1]. The lowered fiber content following fermentation indicates that fibrous tissues become softer and more digestible owing to the activities of the microorganisms responsible for the bioconversion of lignocellulose and carbohydrates into protein. Additionally, the decreased fat content could be caused by oxidation reactions which may occur during fermentation [32]. Fawale et al. [33], showed that the protein and ash contents of kariya (*Hildergardia barteri*) seeds increased, while their carbohydrate content decreased after fermentation [33]. The chemical composition of sorghum cultivars (Tabat and Dabar) slightly changed as the fermentation process progressed. The crude fiber and protein contents rose, whereas the carbohydrate content decreased [34].

Osungbade et al. [35], demonstrated an increase in the content and in vitro digestibility of Sandbox (*Hura* crepitans) seed protein by elevating temperature (up to 100 °C) and fermentation time (24–96 h). The fat content of the fermented samples declined with an increase in the fermentation time, presumably due to the increased fat breakdown into fatty acids and glycerol during the process [35,36]. The crude protein content of sandbox flour was elevated (*p* < 0.05) as fermentation lasted longer, which can be ascribed to net protein synthesis through the fermentation of the seeds [37]. Moreover, the reduced crude fiber content can be attributed to the degrading activity of the fermenting microorganisms [35]. In an investigation performed on the fermentation of Ghanaian cocoa beans by yeast [38], it was understood that fermentation and prolonging the pod storage led to continuous reductions in the ash, fat, and protein contents of the beans, while the carbohydrate content rose. Furthermore, lengthening the pod storage and fermentation time raised the copper content of the beans, whereas decreases were observed in the magnesium and potassium contents. Their findings also revealed that potassium had the highest content among the minerals, followed by magnesium, phosphorus, and calcium in both the unfermented and fermented cocoa beans [38].

## 7. Effect of Fermentation on Nutritional Properties of Proteins

Nutritional properties refer to the characteristics of food components that influence their contribution to human health, including digestibility, bioavailability, and physiological effects. In the context of proteins, fermentation has been widely studied for its impact on nutritional properties such as digestibility, antioxidant capacity, reduction in antinutritional factors, and allergenicity.

### 7.1. Digestibility

Gastrointestinal digestion can be defined as a chemical and physical process in which food is decomposed by various enzymes to release the nutrients necessary for the survival of the organism. Digestible proteins are degraded into a number of peptides and amino acids [39]. Digestibility is a term commonly utilized to assess the proteolysis capability and availability of proteins. The more digestible a protein is, the more and the easier it is absorbed by the human body, due to the availability of more amino acids for absorption and better nutritional value [40]. Highly digestible proteins are even more suitable than those with poor digestibility, as they are high quality, including a large fraction of amino acids accessible for absorption, and provide extra nutritional value. Thus, researchers have always been seeking proper methods to improve the digestibility of proteins, particularly plant-based ones. Fermentation is an adequate technique for achieving biochemical changes, in particular with respect to the bioavailability and bioaccessibility of the food structure. It can result in a reduction in non-nutritive components such as trypsin and enhance the cross-linking of proteins. Furthermore, it improves proteolysis and the production of amino acids from the matrix, thereby enhancing the digestibility of plant-based proteins [18,41]. Several scientists have carried out research on the enhancement of protein digestibility after fermentation, for different sources of proteins (Table 1). For instance, researchers have reported improvements in the digestibility of quinoa protein concentrate [41], and Bambara protein isolate [29].

### 7.2. Allergenicity

A food allergy is characterized as a negative response of the human immune system to a dietary item that is otherwise considered safe [6]. Proteins are commonly the major allergens, either due to the presence of specific epitopes that trigger IgE-mediated immune responses or because of their low digestibility, which allows intact allergenic peptides to reach the immune system [56]. Fermentation has emerged as a promising strategy to reduce protein allergenicity by altering the structural and immunological properties of allergenic epitopes.

Fermentation of egg white protein using *Lactobacillus sakei*, *Lactobacillus sanfranciscensis*, and *Lactobacillus delbrueckii* revealed that *L. delbrueckii* reduced IgE binding by 50%, attributed to structural modifications of ovomucoid, the dominant allergen in egg [57]. These modifications likely involve proteolytic cleavage and conformational changes that disrupt IgE-binding epitopes. Similarly, fermentation of wheat flour with *Lactobacillus brevis* MS-99 lowered IgE binding capacity by up to 80% [58], possibly due to degradation of allergenic proteins and masking of epitopes through glycosylation or aggregation.

Soybean protein fermentation with *Lactobacillus plantarum* reduced IgE binding by up to 96% [59], which may be explained by hydrolysis of storage proteins like glycinin and β-conglycinin, leading to loss of conformational epitopes. Milk protein fermentation with *Streptococcus thermophilus* subsp. *salivarius* and *Lactobacillus acidophilus* diminished IgE binding by more than 70% without intense proteolysis [60], suggesting that subtle conformational rearrangements or epitope masking can also reduce allergenicity.

Solid-state fermentation (SSF) using *Rhizopus oligosporus* altered the proteome of white lupin, decreasing the frequency of allergenic peptides and reducing tryptic peptides derived from β-conglutin, the main allergen [61]. This reduction is likely due to enzymatic cleavage and structural unfolding that disrupt epitope accessibility. Zhou et al. [62], reported a decline in allergenicity of Tartary buckwheat by 39.1% and 82.8% using *Pediococcus pentosaceus* and *Lactococcus taiwanensis*, respectively, presumably due to fermentation-induced conformational changes and partial hydrolysis of allergenic proteins.

Fermentation with *Bacillus natto* reduced peanut protein IgE reactivity by over 77.3% [63], likely through enzymatic degradation of Ara h proteins and alteration of their tertiary structure. Fu et al. [48] demonstrated that wheat protein fermentation with *Pediococcus acidilactici* XZ31 and yeast led to reduced antigenicity of albumin/globulin and increased reactivity of gluten R5, with the most significant changes observed in co-culture, indicating selective degradation and epitope reshaping.

Rao et al. [64] also observed decreased allergenic activity in wheat proteins following fermentation with Chinese traditional starters. Other studies have reported reductions in allergenicity of lupin protein hydrolysate [65], lupin protein isolate [66], bovine whey proteins [67], and cow milk proteins [68], often linked to proteolytic breakdown, conformational changes, and epitope masking or destruction [68].

### 7.3. Antioxidant Activity

Antioxidants are molecules preventing the oxidation of other compounds and are broadly applied in dietary supplements [69]. Fermentation can alter the antioxidant capacity of proteins, due to making changes to antioxidant molecules including vitamin C, glutathione, and tocopherol. Moreover, some of the amino acids and active peptides produced during proteolysis have antioxidant activity [6].

Fermentation has positive effects on the antioxidant activity and total phenolic content of cereals, which depend on the microorganisms used. Lactic acid bacteria are widely utilized in food fermentation [70]. Antioxidant capacity can be quantified using various methods including peroxyl-radical-trapping capacity (PRTC), Trolox-equivalent antioxidant capacity (TEAC), superoxide dismutase (SOD)-like activity, lipid peroxidation inhibition, and reduction of 2,2-diphenyl-1-picrylhydrazyl (DPPH) and 2,20-azinobis-(3-ethylbenzthiazolin-6-sulfonic acid (ABTS) radicals [71]. The enhancement of antioxidant activity by fermentation is primarily owing to the rise in the total phenolic and flavonoid contents caused by microbial hydrolysis. Microbial enzymes hydrolyze phenolic glycosides and produce aglycone which has antioxidant activity. Additionally, fermentation brings about the structural breakdown of plant cell walls, which results in the release or synthesis of different antioxidants [72].

The antioxidant activity, measured by DPPH, OH, and ABTS radical scavenging assays, was found to be significantly higher in wheat gluten fermented with *B. subtilis* compared to the control. This enhancement is likely attributed to the increased presence of diverse peptides.

It was suggested that fermentation using *Bacillus subtilis* altered the structure of the wheat gluten and released some small-molecule active peptides and amino acids with certain electron donor capacities, indicating the reducing power [73].

Gum et al. [74] produced fermented yeast rice product (FRYP) using *Bacillus subtilis*, which had higher free radical scavenging ability and antioxidant activity than the unfermented sample. This could be caused by the large amounts of phenolics and benzofuranone (a transformation metabolite). Nevertheless, more research should be carried out on the structure–activity correlations, properties, and possible working mechanisms of antioxidants (Gum et al., 2017) [74].

Zhang et al. [75] fermented peanut meal using *Bacillus subtilis* and maintained that the resulting peanut peptide had a maximum DPPH scavenging activity of 63.28% at 1.0 mg/mL [75].

Wang et al. [76] ranked the post-fermentation alterations in the antioxidant activity of eight whole grains as follows: millet, wheat, sorghum, black rice, buckwheat pear, rice, black glutinous rice and red quinoa. They declared that the DPPH and ABTS free radical scavenging activities were, respectively, elevated by 239 and 325% after fermentation [76].

The antioxidant activity (ABTS, DPPH, and lipid peroxidation) of whey protein after fermentation with *Lactiplantibacillus plantarum* was enhanced, and the samples induced lipid oxidation to a lesser extent [77]. Soy protein hydrolysates fermented using *Lactobacillus delbrueckii* subsp. *bulgaricus* produced through hydrolysis with cell envelope proteinase (CEP) for 4 h had the highest antioxidant activities, denoted by proper ABTS, DPPH, and hydroxyl radical scavenging activities [78].

Dai et al. [19] investigated the effects of single- and two-stage fermentations using thermophilic *Bacillus licheniformis* YYC4 on soybean protein antioxidant activity and claimed that the two-stage SSF dramatically improved the bioactivity (i.e., reducing power, hydroxyl radical scavenging ability and ACE inhibition activity) of the soybean protein. Fermentation enhanced the antioxidant activity of beverages based on whey protein concentrate [79]. Other studies have also demonstrated improvements in the antioxidant activity of fermented maize protein [80] and plant-based foods [72].

Fermentation conditions strongly influence antioxidant activity by shaping microbial metabolism and enzyme action. Optimal time and temperature typically enhance activity through the release of bound phenolics, conversion of glycosides to more active aglycones, and production of antioxidant peptides and organic acids, while prolonged or extreme conditions may cause degradation [81,82]. The choice of microorganism and fermentation mode is also critical, as lactic acid bacteria, Bacillus species, and filamentous fungi differ in enzymatic capacity, and solid-state fermentation often yields greater increases in antioxidant activity than submerged systems [83,84]. Factors such as pH, inoculum size, and salt concentration further modulate these effects, highlighting the need for condition-specific optimization and the use of multiple assays for accurate evaluation [85].

### 7.4. Antinutritional Compounds

Consumers have continuously shown interest in plant-based diets. Dietary guidelines have always recommended the inclusion of grain legumes and cereals in the diet. At the same time, the presence of antinutritional factors with detrimental effects on nutrition or harmful ingredients, especially in cereals and pulses, decreases their nutritional values by reducing the bioavailability of proteins, vitamins, and minerals [86,87].

Plants normally generate these factors to protect themselves, which limits their applications as food and feed ingredients [88]. Vicine and convicine, α-galactosidase, lupin alkaloids, inhibitors of enzymes like trypsin, chymotrypsin, and α-amylase, polyphenols such as tannins, oxalates, cyanogenic glycosides, lectins, saponins, phytic acid, isoflavones, and biogenic amines are all examples of these antinutritional factors [89].

The human body can slightly absorb oxalates in normal non-fasting conditions. If dietary sources with excess oxalates are consumed, they may be excreted as insoluble calcium salts [90].

Lectins are proteins with a special affinity for specific sugars. They are hemagglutinins (HA); i.e., they are capable of agglutinating red blood cells. Lectins that are given orally to humans and animals are very poisonous. Some substances which naturally exist in plants restrain amylolytic and proteolytic enzymes including amylase, trypsin, and chymotrypsin. Among the inhibitors of proteolytic enzymes, trypsin inhibitors are perhaps the most prevalent. They are resistant to proteolysis and high temperatures [91].

Polyphenols are capable of crosslinking proteins, making them less sensitive to enzymes during digestion, thereby reducing their digestibility. They also possess interesting characteristics including antiallergenic, anti-inflammatory, antiatherogenic, antimicrobial, antithrombotic, antioxidant, vasodilatory, and cardioprotective effects. Phenolic acids, condensed tannins, and flavonoids constitute the major groups of the phenolic compounds of legumes [92].

In natural or pure mixed-culture fermentations of plant-based foods by bacteria, molds, and yeasts, antinutritional agents, such as phytate in whole wheat breads, could be decreased up to 50%, and toxic compounds, e.g., lectins in Tempe and other fermented products produced from beans, could decline by up to 95% [93]. The literature demonstrates the effects of fermentation on the decomposition of antinutritional components and the bioavailability of minerals and proteins [94]. Faba bean, a member of the *Fabaceae* family, is an essential legume that could be applied both as feed and food. It has a noteworthy nutritional profile; however, two toxic glycosides, namely vicine and convicine, naturally occur in its composition.

Several studies have revealed that fermentation caused a decrease in the concentrations of vicine and convicine, which have been proven to play a role in the formation of hemolytic anemia (favism) [95,96]. Some fungi like *Fusarium graminearum* and *Aspergillus oryzae* as well as some *Lactobacillus* strains produce Ɓ-glucosidase participating in vicine breakdown [96]. Fermenting Faba bean flour by *Lb. plantarum* was found to completely decompose vicine during the fermentation [96].

Saponins are glycoside derivatives consisting of a carbohydrate moiety (mono/oligosaccharide) linked to an aglycone and naturally occur in a variety of plants at different concentrations. They are surface-active triterpene or sterol glycosides in which sugars (pentoses or hexoses) are attached to an apolar moiety, called sapogenin, which might be either a triterpene or a sterol [93]. They are normally bitter, bring about foaming in solutions, reduce nutrient availability, and are able to cause hemolysis in red blood cells [97].

At the same time, saponins have health-promoting effects on the human body, including lowering cholesterol levels and risk of cardiovascular diseases [86,98]. Cooking does not destroy them. Nevertheless, fermentation has been shown to decrease the saponin content. Fermentation using *Lb*. *plantarum* reduced saponins by 71% in quinoa proteins [99]. Specific fermenting microorganisms including *Streptococcus* sp., *Lactobacillus* sp., and *Leuconostoc* sp. have α-galactosidase activity that enables them to convert α-galactosides to absorbable mono- and disaccharides [6].

Fermentation of Tempeh lowered the levels of phytates, 3-N-oxalyl-L-2,3-diaminopropionic acid (ODAP), and trypsin inhibitors by 22, 93, and 99%, respectively, while protein bioavailability was elevated by about 25% in grass-pea seeds after fermentation [51]. Fermentation of Bambara nut with *Rhizopus* species alone or in combination decreased antinutrients including tannin (from 0.35 to 0.0249 mg/g), phytate (from 35.20 to 10.7049 mg/g), oxalate (from 1.54 to 0.3949 mg/g), and trypsin inhibitor (from 3.22 to 0.49 mg/g). The lowest contents for the entire antinutritional factors studied were obtained after 72 h of fermentation when a combination of *R. oligosporus*, *R. nigricans*, and *R. oryzae* was employed [100].

There was also a significant decline in the activity of trypsin inhibitor, tannin, and phytate in maize flour after being fermented using natural *S. cerevisiae*, *L. plantarum*, and their co-cultures. The fermented maize flour had greater nutritional value than the unfermented flour, because of the activity of endogenous and exogenous enzymes which were capable of degrading the antinutrients [1].

Fermentation of dry bean (*Phaseolus vulgaris* L.) led to decreases in the phytate, saponins, TIA, tannins, and raffinose oligosaccharides present in the beans [101]. The contents of phytate, alkaloids, tannin, hydrogen cyanide, trypsin inhibitors, lectins, and oxalate declined following the fermentation of *Vigna racemosa*. Open and controlled fermentation shrunk both raffinose and stachyose [102]. Following the SSF of soy protein with *B. subtilis* KCCM11438P, glycinin, β-conglycinin subunits, and trypsin inhibitors decreased by 42, 70, and 50%, respectively, which can be the reason behind the enhanced digestibility of the fermented samples compared with the control [103].

## 8. Effects of Fermentation on Physicochemical and Techno-Functional Properties of Proteins

Functional properties are the physicochemical characteristics of food components, which denote their appropriateness for application in final products. Techno-functional properties, including gelation, foaming, emulsification, and water holding capacity, highly depend on the presence of secondary, tertiary, and quaternary protein structures [104] in addition to the protein electrical charge, molecular weight, and amino acid profile. Changes in these intrinsic structures usually affect the protein flexibility, hydrophobicity, and other functionalities [105].

A food product is considered to be functional if it influences a minimum of one target function in the human body in addition to its proper nutritional impacts, enhances health and well-being, or lowers the risk of attracting a specific disease [106]. Based on this definition, fermented foods can be regarded as functional foods, due to their effects on reducing antinutritional substances and raising protein digestibility and antioxidant activity. Additionally, some specific fermenting microorganisms are considered probiotics. In other words, if they are administrated in sufficient amounts, they will show their health-promoting effects in the host body [107].

Proteins are the basic functional compounds of different high-protein processed foods. As a result, they govern the textural, nutritional, and sensory attributes. Proteins have a variety of functional characteristics, owing to their heterogeneous structure and interactions with other food ingredients [108]. Application of proteins and/or their ingredients in foodstuffs requires that their technological characteristics be optimized. Food proteins have valuable technological properties for various food industries, including water holding capacity, solubility, oil binding capacity, as well as emulsifying, gelling, and foaming properties which fermentation is able to modify, thus developing a broad range of possibilities that depend on the protein type, the fermentation conditions, and the final product properties [6]. Various researchers have demonstrated the modulation of the functional properties of different proteins after fermentation using different microorganism strains (Table 2).

### 8.1. Protein Solubility

Solubility has been recognized as a prerequisite for other techno-functional properties of proteins. As a thermodynamic and one of the most critical functional attributes, it can be defined as the concentration of protein in a saturated solution in the presence of solvent, salts, and other additives, at a specific pH and temperature, in equilibrium with an amorphous or crystalline solid phase [12,120]. Generally, high solubility in aqueous media facilitates the development of protein-rich food systems and enables the manifestation of functional properties such as emulsification, foaming, stabilization, and gelation—attributes that are often unattainable with insoluble proteins.

The factors influencing solubility are broadly categorized into external (e.g., ionic strength, temperature, pH, and additives) and internal (e.g., amino acid composition and distribution on the protein surface) determinants. Among these, pH plays a pivotal role in modulating protein solubility through its influence on net surface charge and conformational stability. Proteins exhibit minimal solubility at their isoelectric point (pI), where the net charge is zero, leading to reduced electrostatic repulsion and enhanced hydrophobic interactions that promote aggregation. At pH values above or below the pI, proteins acquire net negative or positive charges, respectively, which increase solubility by inducing electrostatic repulsion and preventing molecular association.

Furthermore, exposure to acidic conditions (pH < 4) can induce partial denaturation, resulting in the unfolding of tertiary structures and the exposure of hydrophobic domains. While this may reduce solubility due to aggregation, it can simultaneously enhance interfacial activity, thereby improving emulsifying capacity. Ionic strength also modulates solubility by shielding electrostatic interactions and disrupting protein–protein associations, particularly near the pI. These physicochemical changes are especially relevant in fermented protein systems, where processing conditions such as pH and salt concentration are critical for optimizing solubility and functionality.

One major phenomenon thought to influence the solubility of proteins is denaturation. Most proteins exhibit moderate stability at neutral pH and ambient temperature, but are susceptible to denaturation under altered thermal, pH, or pressure conditions, as well as during freeze–drying and freeze–thawing—processes commonly employed in food and pharmaceutical industries [12]. Ultimately, solubility remains a central determinant of protein functionality, directly impacting their ability to emulsify, gel, and foam [121].

### 8.2. Emulsifying Properties

Emulsifying properties of proteins are generally evaluated by the emulsifying activity index (EAI) and emulsion stability index (ESI). EAI represents the capacity and ability of a protein to be adsorbed at the oil-water interface of an emulsion, i.e., the surface area developed per unit mass of the emulsifier [122]. Emulsifying properties are significant functional properties for product development, as they cause proteins to interact with lipids (Jain and Anal, 2017) [109]. In general, proteins have acceptable emulsifying properties, which is why they are usually utilized in food emulsions. Proteins consist of nonpolar amino acids, charged amino acids, and non-charged polar amino acids. This makes them a potential emulsifier, i.e., a surfactant with both hydrophobic and hydrophilic natures, which can interact with both water and oil in food [123]. The emulsifying properties of proteins originate from their amphiphilic character which shrinks the interfacial tension at the oil–water interface in water-in-oil (W/O) or oil-in-water (O/W) emulsions.

At the molecular level, fermentation enhances emulsifying properties by hydrolyzing proteins into smaller peptides with increased surface activity and amphiphilicity, facilitating better adsorption at oil–water interfaces [2].

### 8.3. Foaming Properties

The protein foaming capacity (FC) is quantified by measuring the interfacial area which can be created through whipping the protein mechanically. Foam stability (FS) is defined as the foam volume retained after 30 min. Foam formation and FS depend on the protein type, processing methods, pH, surface tension, and viscosity [118]. They are some of the significant functional properties which should be considered for the application of proteins in food products. Similarly to emulsifying properties, foaming properties are dependent on the characteristics of the protein (capability of migrating to the air–water interface, reducing surface tension, and rearranging its hydrophilic moiety in the direction of the polar phase and its hydrophobic moiety along with the non-polar phase), environmental conditions (the concentration of the protein, temperature, pH, and the presence of other ingredients), and the foam formation conditions [6]. Foaming capacity is improved through fermentation-induced unfolding of protein structures, which exposes hydrophobic domains that stabilize air bubbles [124].

### 8.4. Water Holding Capacity and Oil Binding Capacity

The interaction between proteins and water has interchangeably been stated using different terms, namely water hydration and holding, water binding, water retention, water adsorption, and water imbibing. Water holding capacity is the ability of a food ingredient to hold its own and added water during different processes such as pressing, heating, or centrifugation. The degree of protein hydration is interrelated with the viscosity of liquids in food products. It is one of the hydration characteristics which influence protein applications in food systems. Water holding capacity is defined as the ability of a material to maintain water against gravity physically and physicochemically. Many of the functional attributes of proteins pertain to their interaction with water. Therefore, protein-water interactions including water binding and retention, solubility, swelling, emulsifying properties, gelation, viscosity, and syneresis, affect the functional properties of proteins in food systems, [125].

The significance of fat binding as a functionality is dependent on the food type, for example, fluid emulsions, dairy products, powders, sausages, dough and bread. The binding of fat with food ingredients, especially carbohydrates and proteins, affects the quality attributes of foods. The fat absorption and retention capability of proteins as well as their ability to interact with lipids in food systems is notable in food preparations [126]. Numerous food properties such as emulsion formation, fat entrapment in sausage batters, fat emulsification in meat, dough preparation, and flavor absorption depend on the protein-lipid interactions. The fat absorption ability of proteins is determined by the source of the protein, composition of additives, processing conditions, temperature, and particle size. Fat binding ability is intensely correlated with the food protein content, which is mainly regarded as the physical entrapment of oil by chemically modifying the proteins, which raises their bulk density [125]. Fermentation affects WHC and OBC through structural modifications. Partial hydrolysis exposes polar groups (e.g., –OH, –COOH, –NH_2_), increasing the number of hydrogen-bonding sites available for water retention. Similarly, unfolding of protein structures exposes hydrophobic residues, enhancing lipid interaction and oil entrapment. Increased surface area and solubility of peptides also facilitate stronger hydration shells and improve fat absorption. Conversely, extensive proteolysis may reduce the ability to form three-dimensional networks, leading to lower WHC and OBC. The effects of fermentation on the water holding capacity and oil binding capacity of proteins are summarized in Table 2.

### 8.5. Gelling Properties

The gelling capacity of proteins is a key functional characteristic for food manufacturers. Gels are essential foods, in which proteins are the gelling ingredients. They create resistant gels when accompanied by pectins, gums, and starches. Different food industries employ various proteins to manufacture gels or gel-containing products which show different rheological properties, gel points, and appearances. The gel-forming ability of proteins affects their other functional properties such as oil binding capacity and water retention. The gelation phenomenon has a substantial effect on stabilizing foams and emulsions. Enzymatic hydrolysis exposes sulfhydryl and hydrophobic groups that participate in disulfide bonding and hydrophobic interactions, which are critical for gel network formation. Mild fermentation improves gelation by producing peptides that are flexible and interact readily, while extensive degradation can weaken the protein network, leading to softer or less cohesive gels. Changes in peptide size distribution and charge density during fermentation also affect water entrapment and elasticity of the gel matrix.

## 9. Effect of Fermentation on the Sensory Attributes of Proteins

Fermentation significantly enhances the sensory quality of protein isolates and hydrolysates by reducing undesirable attributes such as bitterness, astringency, and the characteristic “beany” flavor common in legume proteins. These improvements are driven by enzymatic hydrolysis and microbial metabolism, which modify volatile compounds and generate taste-active molecules [121,127,128].

The beany flavor is primarily caused by volatile aldehydes and ketones, especially hexanal, 1-hexanol, 2-pentylfuran, and octen-3-one which are formed through lipoxygenase-mediated oxidation of polyunsaturated fatty acids like linoleic acid. During fermentation, microbial strains such as *Lactobacillus plantarum*, *Zygosaccharomyces rouxii*, and *Bacillus subtilis* degrade these compounds via specific enzymatic pathways. For instance, alcohol dehydrogenase (ADH) reduces hexanal to hexanol, while aldehyde dehydrogenase (ALDH) oxidizes hexanal to hexanoic acid, which can further react to form esters like ethyl hexanoate, contributing fruity notes. Additionally, microbial esterification and lactone formation produce pleasant aroma compounds such as γ-nonalactone and β-damascenone, enhancing the overall flavor profile [129,130,131].

Quantitative sensory evaluation has been conducted using a combination of quantitative descriptive analysis (QDA) and hedonic testing. Trained panelists assessed attributes like bitterness, umami, and aroma intensity using 15 cm unstructured line scales, while consumer panels rated overall acceptability on a 9-point hedonic scale. Statistical analysis (ANOVA, Tukey’s HSD) confirmed significant improvements (*p* < 0.05) in fermented samples.

For example, fermentation of soy protein hydrolysates with *Tetragenococcus halophilus* and *Zygosaccharomyces rouxii* increased umami amino acids by 30.6% and sweet amino acids by 62.0%, correlating with higher sensory scores [129]. Fermentation of pea protein isolate reduced aldehyde and ketone levels by 42% and 64%, respectively, improving aroma and reducing beany flavor [130]. Similarly, *Lactobacillus plantarum* fermentation significantly reduced bitterness (*p* < 0.001), raising hedonic scores from 5.2 to 7.1 [131].

Volatile compound analysis confirmed the removal of hexanal and other off-flavor contributors during fermentation with *Lactobacillus* and *Streptococcus* species [132]. GC-MS profiling revealed a 27.36% reduction in aldehydes and a 10.96% increase in ketones and esters, contributing to a more balanced and pleasant aroma. Additionally, fermentation reduced particle size from 142.4 μm to 122.7 μm, improving mouthfeel and reducing astringency [65]. These molecular changes, validated through structured sensory evaluation and volatile profiling, demonstrate fermentation’s effectiveness in improving the flavor and acceptability of plant-based protein ingredients.

## 10. Conclusions and Future Trends

This study highlights fermentation as a transformative approach for enhancing the nutritional, functional, and sensory properties of protein isolates and hydrolysates. Through microbial metabolism and enzymatic modification, fermentation improves solubility, modulates surface charge and hydrophobicity, and enhances water and oil retention, emulsifying, foaming, and gelling capacities. Importantly, it also reduces undesirable sensory attributes such as bitterness and beany flavor, while increasing umami and sweetness—making fermented proteins more suitable for diverse food applications.

The extent of these improvements is influenced by the type of protein (e.g., legume, dairy, cereal), the form of the substrate (flour, concentrate, isolate), and the fermentation parameters (microorganism strain, temperature, pH, duration). These variables must be carefully optimized to achieve targeted functional and sensory outcomes.

Looking ahead, future research should focus on the following:

Mechanistic studies to map the molecular pathways of flavor compound degradation and protein structural changes during fermentation.

Predictive modeling to design fermentation protocols tailored to specific protein sources and desired functionalities.

High-throughput screening of microbial strains for targeted improvements in emulsification, gelation, and flavor enhancement.

Application-specific formulation of fermented proteins in plant-based dairy analogs, meat substitutes, baked goods, and high-protein beverages.

Integration with other processing technologies (e.g., enzymatic hydrolysis, extrusion, Maillard reaction) to synergistically enhance protein quality.

Ultimately, fermentation offers a sustainable and versatile platform for developing next-generation protein ingredients that meet consumer demands for nutrition, taste, and environmental responsibility. A knowledge-based design approach—where fermentation parameters are adapted to achieve specific product goals—will be essential for advancing protein innovation in food systems.

## Figures and Tables

**Table 1:** The effects of fermentation on the digestibility of various protein sources.

Protein Source	Fermentation Organism	Digestibility	Ref.
**Milk proteins**			
Whey protein	Water kefir (including lactic and acetic acid bacteria and yeast)	It could enhance the protein digestibility of fermented whey proteins from 88.48 ± 0.94 (unfermented) to 94.33 ± 2.05% on Day 5	[42]
Whey protein concentrates	*Streptococcus thermophilus* RBC6, RBC20, and RBN16	Fermentation enhanced the hydrolysis of WPC proteins that had a positive impact on gastro-intestinal digestion	[43]
**Cereal proteins**			
Sorghum flour supplemented with whey protein	Natural fermentation	The in vitro protein digestibility was significantly improved during fermentation and even after supplementation	[44]
Sorghum protein	Natural and *Lactobacillus plantarum* fermentation	Fermentation leads to increase in vitro protein digestibility (IVPD) by 46.89% and 92.08%, for natural and *L. Plantarum*, respectively. This is associated with hydrolysis of protein and tannin in sorghum	[45]
Sorghum protein	Yeast (*Saccharomyces cerevisiae*) and, *Lipomyces kononenkoae*	Increased in pepsin digestibility of sorghum protein compared to thermally processed control samples	[46]
Wheat Protein in Sourdough and Crackers	*Lactobacillus plantarum* HLJ29L2 and Yeast	There was a strong increase in protein digestibility after fermentation	[47]
Wheat protein	*Pediococcus acidilactici* XZ31 and yeast	Co-culture fermentation with *Pediococcus acidilactici* XZ31 and yeast led to improvement in digestibility of wheat protein compared to single strain fermentation	[48]
**Legume Proteins**			
Lentils, chickpea, rice and wheat	Natural fermentation	Digestibility was 45.30%, 58.76%, 80.3% and 82.6% for raw lentils, chickpea, rice and wheat and became 84.18%, 85.07%, 88.27%, 87.12%, respectively, at the end of the fermentation period	[49]
Flour and whole bean seeds (*Phaseolus vulgaris*)	Natural fermentation	Desirable increase in digestibility because of fermentation	[50]
Grass-pea seeds	*R. oligosporus* DSMZ 1964	Considerable increase in protein bioavailability of grass-pea seeds (higher in the case of cooked ones) which could be due to both thermal denaturation and elimination of anti-nutrients	[51]
Pea protein concentrate	*Lactobacillus plantarum*	Protein digestibility reached a maximum (87.4%) after 5 h of fermentation; however, the alteration of sulfur amino acid content resulted in an overall reduction in protein quality	[27]
Lentil (*Lens culinaris*) proteins	Using water kefir seed	Protein digestibility increased from 76.4 to 84.1% over the 5 days of fermentation.	[52]
*Zamne* (*Senegalia macrostachya* seeds)	*Rhizopus oryzae*	Improving the protein hydrolysis degree which led to increase in digestibility	[53]
Chickpea protein	*Lactobacillus Plantarum* HLJ29L	Improving the hydrolysis of protein during gastric and intestinal digestion by altering the multilevel structures of chickpea protein	[40]
Red kidney bean protein	*Rhizopus oligosporus* RT-3	Solid-state fermentation facilitated the structural breakdown of cotyledon cell walls, thereby further improving protein digestibility	[54]
**Other Plant proteins**			
Sandbox (*Hura crepitans* seeds)	Natural fermentation	Fermentation and cooking of *H. crepitans* seeds improved their protein contents and in vitro digestibility	[35]
Rapeseed protein	*Bacillus subtilis* strain 87Y	Improving the ileal digestibility of protein, crude fat and crude fiber (by about 4%, 6% and 14%, respectively) and significantly improved the digestibility of micro- and macro-elements	[55]

**Table 2:** The effects of fermentation on the techno-functional properties of various protein sources.

Protein Source	Fermentation Organism	Functional Properties				
		Protein Solubility	Emulsifying Properties	Foaming Properties	Surface and Bulk Properties	Ref
**Animal and Milk proteins**						
Protein hydrolysates from eggshell membranes	*Lactobacillus plantarum*	Improvement of solubility	Increase in emulsifying activity	Increase in foaming capacity	-	[109]
Protein hydrolysates from broiler chicken gizzards	*Bifidobacterium longum* B379M and *Propionibacterium freudenreichii*	Improvement of solubility	Increase in emulsion capacity	Increase in foaming capacity	Increase in WBC Increase in OBC	[110]
Bovine beta-lactoglobulin and hen egg ovalbumin	*Trichoderma reesei*	-	Improvement in emulsion stability and emulsifying capacity	Increase in foaming capacity	Improvement in heat- gelation	[111]
Whey protein	Kefir	Improvement of solubility	-	-	Decrease in surface hydrophobicity	[42]
Whey protein concentrates	Yeast (*Saccharomyces cerevisiae*)	Improvement in solubility	Enhancement in the emulsifying activity and stability	-	increase in surface hydrophobicity	[28]
**Plant proteins**						
Cowpea (*Vigna unguiculata*) protein	*Rhizopus microsporus* subsp. *oligosporus*	Increase in solubility	Higher emulsion capacity	-	-	[112]
Sorghum flour protein	Traditional Sudanese method	Shifting in the solubility of sorghum proteins by 2 pH units and more soluble protein at pH isoelectric	Increase in emulsifying/capacity (EC) and emulsifying stability (ES)	-	Increase in OBC Decrease in gelation concentration Decrease in WBC	[113]
Lupin seeds protein	*Leuconostoc mesenteroides*, *Lactobacillus plantarum* and *Lactobacillus brevis*	Increase in soluble protein content	Decrease in emulsifying activity	-	Increase in WAC and WHC	[114]
*Mucuna cochinchinensis* protein isolate	Natural fermentation	-	Increase in emulsifying capacity	Increase in foam capacity and foam stability	Decrease in OBC Decrease in WBC Increase in gelation capacity	[115]
Peanut protein concentrate	*Rhizopus oligsporus*	Increase in solubility of defatted roasted peanut flour across the pH range tested (pH 3.0–10.0)	Increase in emulsifying capacity	Increase in foam capacity	-	[116]
Sandbox (*Hura crepitans*) seeds protein	Natural fermentation	Improvement of protein in alkaline pHs	Decrease in emulsifying properties	Decrease in foaming capacity	Decrease in OBC Decrease in WBC Increase in least gel concentration	[35]
Lupin protein isolate	8 different lactobacillus strains	Decrease in solubility at pH = 7	Decrease in emulsifying capacity	Increase in foam activity	-	[66]
Bambara protein isolate	-	Improvement in solubility and protein yield	Increase in emulsion stability and capacity	Decrease in foam stability	Decrease in WBC Increase in OBC Increase least gelation concentration	[29]
Pea protein isolate	*L. plantarum*, *L. fermentum*, *L. perolens*, *L. casei*, *Lc. Cremoris*, *P. pentosaceus*	Improvement in protein solubility at pH 5.0 but a significant decrease at pH 3.0	Decrease in emulsifying capacity	No change	-	[117]
Lupin Protein Hydrolysates	*Lactobacillus sakei ssp. carnosus*, *Lactobacillus amylolyticus and Lactobacillus helveticus*	Improvement of solubility	Decrease in emulsion capacity and activity	Increase in foaming capacity and stability	-	[65]
Lentil–Casein Protein Complexes	Kefir-Assisted Fermentation	Improvement of solubility	-	-	-	[18]
Pea protein-enriched flour	(*Aspergillus oryzae* NRRL 5590, *Rhizopus oryzae* NRRL 395, *Rhizopus oligosporus* NRRL 2710, *Lactobacillus plantarum* NRRL B4496, and *Bacillus subtilis* ATCC 6051)	Decrease in solubility	Increase in emulsion stability	Decrease in foam capacity (FC) and foam stability (FS)	Increase in WBC Increase in OBC	[118]
Chickpea aquafaba protein	*Lactobacillus plantarum* MA2	Improvement in solubility	Increase in emulsifying capacity	Improvement in foaming expansion and stability	Increase in OBC	[119]

## Data Availability

No new data were created or analyzed in this study. Data sharing is not applicable to this article.
